# Observations on Hilltopping in Thick-Headed Flies (Diptera: Conopidae)

**DOI:** 10.1673/031.010.2701

**Published:** 2010-03-29

**Authors:** Maurizio Mei, Joel F. Gibson, Jeffrey H. Skevington

**Affiliations:** ^1^ Università di Roma “Sapienza”, Dipartimento di Biologia Animale e dell'Uomo, Entomologia, P.le Valerio Massimo, 6, 00162, Roma, Italia; ^2^ Agriculture and Agri-Food Canada, Canadian National Collection of Insects, Arachnids and Nematodes, 960 Carling Avenue, Ottawa, ON, KIA 0C6, Canada; ^3^ Department of Biology, 209 Nesbitt Building, Carleton University, 1125 Colonel By Drive, Ottawa, ON, KIS 5B6, Canada

**Keywords:** Canada, *Dalmannia*, Italy, mating behaviour, *Myopa*, parasitoid, phenology, *Physocephala*, *Thecophora*, *Zodion*

## Abstract

Direct observations of hilltopping behaviour in the thick-headed flies (Diptera: Conopidae) have only been mentioned once in the literature. Hilltop collecting, however, may be an effective way to survey these endparasitoids. The first evidence of hilltopping in species belonging to the subfamilies Myopinae and Dalmanniinae is presented and discussed. Field observations were conducted on Colle Vescovo, Italy and Mount Rigaud, Canada, and museum specimens were examined. Observations and records indicate that four species in the genera *Dalmannia, Myopa,* and *Zodion* are hilltoppers on Colle Vescovo, while three species in the genera *Myopa* and *Physocephala* are hilltoppers on three hilltops near Ottawa, Canada. Fifteen additional species of conopids have been collected on hilltops and could possibly utilize hilltops in some years as a part of their mating strategy. Detailed phenologies and observations of mating and perching behaviours are given for species in the genera *Dalmannia, Myopa, Physocephala,* and *Zodion.* The importance of hilltop habitat preservation is stressed.

## Introduction

Hilltopping in insects, as initially defined by Shields (1967), is the behaviour of males aggregating on a peak to wait for the females that fly there and mate with them. In a paper dealing with some Conopidae from South Africa, Smith (1967) reported an observation made by Stuckenberg on hilltopping behaviour in a species of *Pleurocerinella.* Stuckenberg described a small swarm of these flies, including both males and females, that were found clustering tightly together on a leaf “on the flat summit of a mountain”. He suggested that this behaviour could have had a sexual function. Gathering of Conopidae at a particular landmark, though not on a hilltop, also has been reported by Kröber (1927). Hundreds of *Leopoldius coronatus* (Rondani) of both sexes were observed consecutively for a few days in Lautembach, a site near Gernsbach (Schwarzwald, Germany), on a forest path that was in the sun in the late afternoon. The *L. coronatus* were flying together along a small ditch filled with ferns and brambles but otherwise devoid of flowers: “a place where I could have never expected to find conopids” (Kröber 1927, in translation). Although matings were not described, a sexual function of the gathering is possible.

The above mentioned observations, both relevant to species of the subfamily Conopinae, seem to be the only published evidence of hilltopping and swarming behaviour in this family of flies. Despite this, the phenomenon has been recognized for some time. Label data on specimens in collections clearly indicates that some entomologists have noticed and collected these insects on hilltops for decades. Recently, Skevington (2008), reviewing the available knowledge, suggested that hilltop collecting may be an effective way to survey many groups of insects, particularly parasitoids such as Conopidae, Pipunculidae, and Tachinidae.

This paper provides the first evidence of hilltopping in species belonging to the subfamilies Myopinae and Dalmanniinae. Field observations and museum specimen examinations were conducted in Italy and Canada. This research adds new observational data on hilltopping and mating behaviour in these poorly understood parasitoid Diptera, and will be of interest to those studying insect mating systems, parasitoid-host interactions, and hilltopping.

## Materials and Methods

### Colle Vescovo observational data

**Figure 1.**
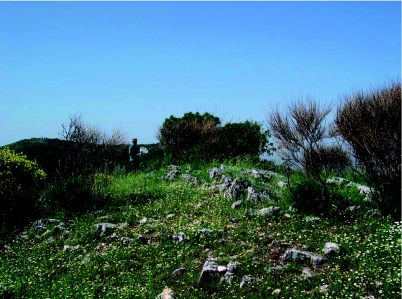
Colle Vescovo, (Latium, Italy, 41° 58′ 39″ N, 12° 48′ 30″ E), summit viewed from the northeast. Picture taken in late May 2008; the traces of a recent fire are still visible, and the 3 m *Pistacia* tree has been removed. High quality figures are available online.

Observations by the senior author took place at the summit of a hill named Colle Vescovo, (448 MASL), at the southwest edge of the Lucretili Mountains near the town of Tivoli (Latium, Italy) (41° 58′ 39″ N, 12° 48′ 30″ E). The hill was covered by very degraded xerophilic bushes, characterized by *Ampelodesma mauritanica* Durand and Schinz (Poales: Poaceae), *Styrax officinalis* L. (Ericales: Styracace), *Paliurus spina-christi* Miller (Rosales: Rhamnaceae), *Pistacia terebinthus* L. (Sapindales: Anacardiaceae), *Cercis siliquastrum* L. (Fabales: Fabaceae), *Phillyrea latifolia* L. (Oleales: Oleaceae), and *Spartium junceum* L. (Fabales: Fabaceae) (Montelucci 1947). There were abandoned olive groves at the foot of the hill and a cork oak thicket on the northeast slope. The summit of the hill was almost bare, with a low rock outcropping, oriented along a northeastsouthwest direction and supporting a large olive bush, *Olea europeea* var. *oleaster* L. (Oleales: Oleaceae) about 2.5 m tall, two much smaller terebrinth, *P. terebinthus,* bushes, and a Spanish broom, *S. junceum* bush. At the time of the observations, a dead, contorted trunk of a small *P. terebinthus* tree, about 3 m tall, was standing in the middle of the larger of the *P. terebinthus* bushes. The hilltop area was approximately 40 square meters (Figure 1). The vegetation of Colle Vescovo and the neighbouring hills was degraded by fires that occur in the surroundings of Tivoli almost every summer, and from overgrazing by cattle and horses. A fire destroyed the vegetation of the northwest slope almost up to the summit in the late summer of 2001.

Observations on Colle Vescovo were carried out from 1999 to 2003. Throughout this period, the site was sampled at irregular intervals from February to August. In all, 30 visits were made to the hilltop for a total of about 100 hours of observation. Research was concentrated in the spring, and most of the observations were carried out in the months of March, April and May (*n* =20 visits). Observations were made primarily in the morning (*n* ≈ 80 hours), from 09:00 until 13:30, when all insects, and conopids in particular, were most active. About half of the total field work was carried out in 2001, when the site was sampled 2–3 times every month from February to May, then monthly until August (*n* =14 visits). The prevailing winds on the summit, as recorded during the course of the observations, were either from the west or southeast.

None of the conopids observed on the hilltop were marked. Due to the relatively sporadic nature of their presence and to the sparse vegetation of the hilltop, however, it was often possible to record the activity of individuals without interruption for many minutes. Specimens were collected with a hand net and were deposited in the senior author's collection. Voucher specimens are in the Museum of Zoology of the University of Rome “Sapienza”.

### Mount Rigaud observational data

**Figure 2.**
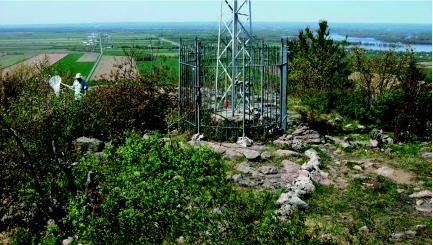
Summit of Mount Rigaud (Quebec, Canada 45° 27′ 59″ N, 74° 19′ 35″ W), looking toward the northwest. High quality figures are available online.

Mount Rigaud is a well-defined hill south of the Ottawa River in southwestern Quebec, Canada (45° 27′ 59″ N, 74° 19′ 35″ W). The hill is in a 5440 ha forested area that was dominated by sugar maple, *Acer saccharum* Marshall (Sapindales: Sapindaceae) and American beech, *Fagus grandifolia* Ehrh. (Fagales: Fagaceae). This area of Quebec has a mosaic of farmland and forest, but the location of the hilltop in a large tract of accessible forest made it one of the most entomologically diverse hilltops in eastern North America (Skevington 2008). The summit of Mount Rigaud is rocky and largely open, with two thickets of stunted northern red oak, *Quercus rubra* L. (Fagales: Fagaceae) (maximum height about 5 m) and shorter thickets of shadbush, *Amelanchier humilis* Wiegand (Rosales: Rosaceae), pin cherry, *Prunus pensylvanica* L., and chokecherry, *Prunus virginiana* Pardee. A viewing stand and a large cross have been built on the summit, but apart from these structures the vegetation is still intact (Figure 2). Skevington visited this site 18 times between May and August from 2001 to 2008. Behaviour of individual flies was observed directly and captured specimens were collected with a hand net and deposited in the Canadian National Collection.

### Data from insect collections

All data from Conopidae collected within 120 km of Ottawa, ON, Canada and represented in the Canadian National Collection of Insects, Arachnids and Nematodes are summarized. Ottawa was chosen as a focal point because three hilltops in the vicinity have received annual collecting attention since 1961. The hilltops involved are King Mountain, QC (49° 29′ 20″ N, 75° 51′ 45″ W), Duncan Lake vicinity, Masham, QC (45° 40′ 53″ N, 76° 3′ 1″ W) and Mount Rigaud, QC. The collections in the Museum of Zoology of the University of Rome “Sapienza” and in the Civic Museum of Zoology of Rome, were examined as well, to summarize data on any conopids collected within 1.5 km of the summit of Colle Vescovo.

## Results

### Colle Vescovo collection records

Fifteen species of Conopidae were collected on the summit and/or the slopes of Colle Vescovo (Table 1), but only four of these were clearly hilltoppers as they were recorded regularly at the summit over the course of the study: *Dalmannia aculeata* L., *Myopa pellucida* RobineauDesvoidy (= *M. extricata,* see Stuke and Clements 2005), *Myopa picta* (Panzer), and *Zodion cinereum* F. (= *Z. notatum*, see Mei and Stuke 2008). Four species were represented by isolated specimens on the summit, and the remaining seven species were not collected on the hilltop (Table 1).

### Ottawa area collection records

Nineteen species of Conopidae were collected in the Ottawa area (Table 2). Three of these are regularly found on hilltops and are rarely found away from hilltop sites: *Myopa clausa* Loew, *Physocephala marginata* (Say), and *Physocephala sagittaria* (Say). A further four species have been collected regularly on hilltops, in addition to non-hilltops: *Dalmannia nigriceps* Loew, *Myopa vesiculosa* Say, *Zodion fulvifrons* Say, and *Zodion intermedium* Banks. Six species have been collected on hilltops, but more evidence is needed to confirm hilltopping as a mating strategy for these species: *Dalmannia vitiosa* Coquillett, *Myopa virginica* Banks, *Physocephala furcillata* (Williston), *Physocephala texana* (Williston), *Thecophora occidensis* (Walker), and *Thecophora nigripes* (Camras). The remaining six species have no records of males collected on hilltops and likely are not hilltoppers: *Myopa vicaria* Walker, *Thecophora abbreviata* (Loew), *Thecophora longicornis* (Say), *Thecophora propinqua* (Adams), *Zodion abitus* Adams, and *Zodion americanum* (Wiedemann).

**Table 1.**
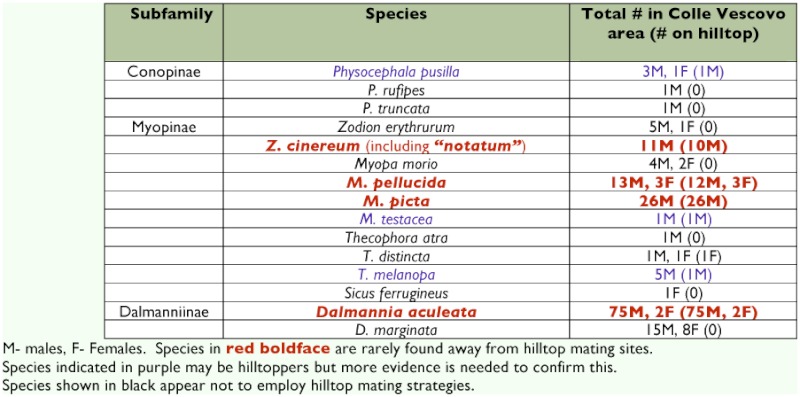
Specimens of Conopidae collected in the study area of Colle Vescovo, noting those collected on the hilltop.

### Phenology

Conopids were found at Colle Vescovo on 11 out of 30 visits, and in general their occurrence on the summit was rather sporadic. The two species of *Myopa* appeared on the hilltop by the end of March (*M. picta,* 24 March 2001, the earliest record) and were recorded at the study site through the first half of May, being most frequent in late April. *D. aculeata* was recorded from the second half of April to the second half of May, although it was particularly abundant around the first week of the latter month. The relatively few available records for *Z. cinereum* were concentrated between the last days of April and the first week of May. By the end of May, none of the four species was still active on the hilltop. Conopids were never observed at the study site in the afternoon. *D. aculeata* was by far the most abundant and frequently recorded species; more than one hundred individuals have been observed, and 75 males and 2 females were collected. About 35 specimens of *M. picta* were observed, and 26 collected, all males. In all, 3 females and 12 males of *M. pellucida,* and 10 *Z.* *cinereum* males were observed and collected. Many *D. aculeata* and nearly all the *Z. cinereum* males collected were newly emerged individuals, with the ptilinum still partly everted.

Conopids were found at Mount Rigaud on most visits. The earliest specimens collected were a male *Z. fulvifrons* on 13 May 1987 and a male *P. texana* on 13 May 2008. The latest specimen taken was a male *Z. americanum* on 25 September 1985. The peak of the diversity on Rigaud was from late May to the end of June. Only two species seem to be common later in the season: *P. furcillata* and *P. sagittaria* typically occur in July and August.

**Table 2.**
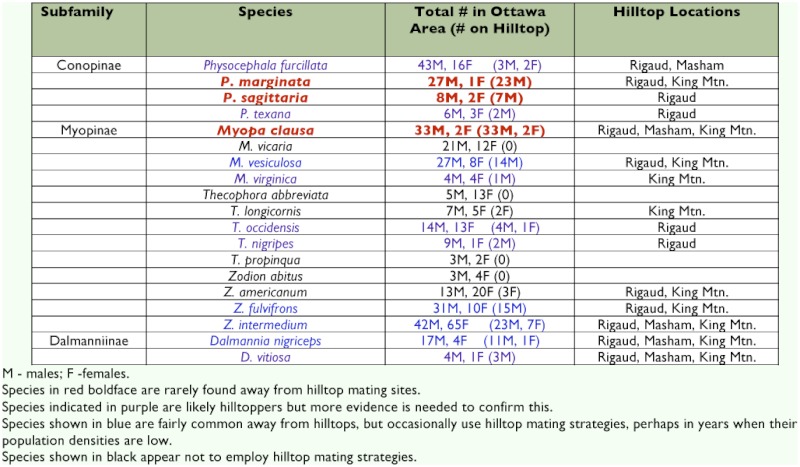
Specimens of Conopidae (Diptera) from the CNC collected in the Ottawa area, noting those collected on hilltops (see text for coordinates of hilltop locations).

### Perch site preferences

The four hilltopping species at Colle Vescovo showed marked preferences in the choice of perch site, and the preferences were strongly consistent from year to year.

*D. aculeata* individuals were invariably observed perched on exposed leaves or on outer twigs of the *O. oleaster* and especially on the *Spartium* bushes, about 0.6 to 1.5 m above ground. Particular (often more prominent) twigs were clearly preferred as perching sites and were regularly occupied over the course of the day(s). On a single occasion a male was collected feeding on a dandelion flower, *Taraxacum* sp. (Asterales: Asteraceae), at the base of the *Spartium* bush.

Most of the *Myopa* individuals were observed in a sheltered spot by the rock, between the *O. oleaster* and one of the *P. terebinthus* bushes, where they chose the lowest twigs of the bushes, the grass stems, and sometimes the flowers of *Taraxacum* and *Bellis* (Asterales: Asteraceae) as perching sites. Such perches were situated from a few centimeters to 0.4 m above ground. Only 2 of the 35 *M. picta* males observed and 3 of the 12 *M. pellucida* were found perching on twigs at the top of the *Spartium* bush, and only once a male of *M. pellucida* was observed feeding on the *Taraxacum* flowers.

All of the *Zodion* males were collected perching and feeding at ground level, on *Taraxacum,* with the single exception of a male that was observed perching on a twig at the top of the *Spartium* bush.

**Figure 3.**
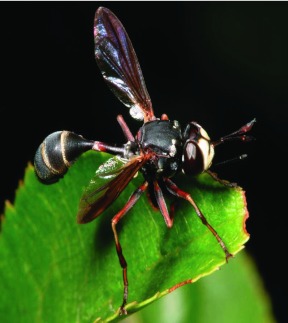
*Physocephala marginata:* a hilltopping species of Conopidae found on Mount Rigaud, Quebec. High quality figures are available online.

Most of the conopids were invariably found on the sides of the bushes and of the summit, directly exposed to the sun. The sun appeared to be the single most important condition to insect activity on the hilltop. Insects, including conopids, were still active in moderately strong winds, provided there was sunlight on the summit; however, activity decreased dramatically and suddenly as soon as the sun was obscured by clouds. In persistently cloudy weather, conopid activity was always nearly absent, and a specimen was only occasionally collected on the summit in such conditions. Sunlight appears to be important limiting factor for all hilltopping Diptera (McAlpine and Munroe 1968; O'Hara 2003; Skevington 2008).

**Figure 4.**
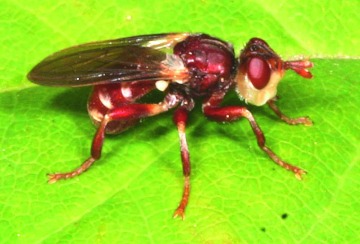
*Myopa clausa:* a hilltopping species of Conopidae found on Mount Rigaud, Quebec. High quality figures are available online.

Notes were made on only a few of the species that occured at Mount Rigaud. *P. marginata* individuals (Figure 3) were almost restricted to perching on a single *P. virginiana* bush (and, in fact, to a small area of exposed leaves about 1.0 m above the ground). This shrub was at the summit of the hill between the viewing platform and a patch of *Q. rubra.* The site was in full sun for most of the day and was one of the most protected spots (i.e., lowest wind) on the summit. Specimens collected were generally replaced within five minutes by another specimen. *M. clausa* (Figure 4) males seemed to use a larger variety of perching sites and were typically closer to the ground. They were often on the *A. humilis* leaves and appeared to readily use sites in both direct sun and dappled light. Individuals of *D. nigriceps* have been collected at Rigaud by Monty Wood of the Canadian National Collection (not all specimens were available to us). He noted that this species was collected by sweeping the topmost branches of the *Q. rubra* (about 5 m above the ground) (Wood, personal communication). During a visit to Rigaud in June 2008, it was found that the largest *Q. rubra* had been pruned of nearly all branches and that males of *D. nigriceps* were now found perching on leaves of a nearby *P. virginiana* shrub at a height of 1.5 m.

### Male behaviour at perch site (observations from Colle Vescovo only)

**Figure 5.**
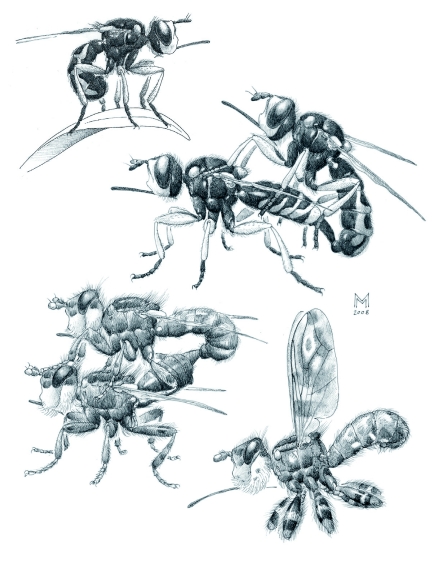
Hilltopping Conopidae from Colle Vescovo, Italy. Top left: *Dalmannia aculeata* seen perching on a leaf. Top right: A male and female *D. aculeata* observed *in copula* under artificial conditions. Bottom left: A male and female *Myopa pellucida in copula* on a *Taraxacum* flower. Bottom right: *Myopa picta* seen in a motionless posture after capture. All images are based on field sketches and “camera-lucida” drawings of prepared specimens. High quality figures are available online.

Waiting *D. aculeata* males (Figure 5) were extremely alert, often adjusting their position on the perch or moving from one perch to another nearby at irregular intervals. It was possible to see their heads frequently tilting to follow the flight of insects, passing by within a distance of about 20–30 cm. Males alternated periods of perching with brief patrolling flights about the bush in which the perch was located, at a distance of a few centimeters from the bush itself. Some of these patrolling flights, however, were much longer with the insect leaving the bush to an unknown location. Sometimes such reconnaissance flights appeared spontaneous, but often they were triggered by the approach of a flying, similar-sized insect (e.g., tachinid fly or small bee). In such a case, the conopid would suddenly follow and closely inspect the insect, returning soon after to the bush.

The approach of a conspecific male initiated more complex interactions, involving apparently ritualized flight contests or actual contact among the opponents, often in the form of pseudocopulation. A perching *D. aculeata* male would dart toward an approaching conspecific and a frantic skirmish would follow, with both flies flying, making loops and spirals in the air while keeping within a few centimeters of each other, and always facing the bush at a short distance from it. This kind of interaction could last for 20 s to more than 1 min at a time (*n* = 6 interactions observed). The chase could stop abruptly, the two combatants going to rest on nearby perches only to resume the contest after a while. During such a flight one of the flies would grasp the other, and they would fall together almost to the ground before parting again. In one occasion, three *D. aculeata* males were involved at the same time in such a chase.

Pseudo-copulation was observed quite frequently between males (*n* = 10). Most of these instances consisted of a male approaching a perching conspecific male, facing it briefly, and then pouncing on it, apparently trying to copulate. Mounted on its dorsum, the attacker extended the abdomen downwards in an attempt to clasp its genitalia. Most often the attacked males initially did not even try to dislodge the intruder; instead, on two instances, the attacked male was observed raising its abdomen like a female during copulation (see below). On one occasion, however, the perched male reacted to the attack, and both males fell together toward the ground. Pseudo-copulation could also end an aerial chase, with the clutched individuals suddenly landing on the bush, where one of them tried to copulate with the other. At irregular intervals, all perching males would perform self-grooming (see below), most often soon after a patrolling flight or a chase.

There were fewer observations of *M. picta* and *M. pellucida.* Males of both species were observed alertly perching on convenient perch sites, chasing conspecifics and other insects, like hoverflies, tachinids and small bees. Pseudocopulation was observed once for *M. picta:* what seemed to be a mating pair perched on a grass stem, was netted and confined in a vial, where it turned out that two males were involved. The flies remained attached for more than 30 min.

*Z. cinereum* males were observed waiting on flowers and on the *S. junceum* bush, but the few encounters with this species on the summit did not allow for more detailed observations.

### Observations on mating behaviour (observations from Colle Vescovo only)

A pair of *D. aculeata* was observed at 09:45 on 12 May 2003, perched on a twig of the *S. junceum* bush. The male, a newly emerged individual, was standing on the dorsum of the female, but the genitalia were not engaged: it was assumed that the copulation had already occurred (see below). After being carefully placed into a glass vial, the pair eventually separated and afterwards the two individuals did not interact. On a second occasion an actual copulation of this species was observed under artificial conditions: a male and a female separately collected on the hilltop during the morning of 1 May 2002, were put together (in the afternoon) in a large glass vial stopped with cotton wool that contained a strip of paper as support. As soon as the male came in contact with the female, it grasped the female from behind and extended its genitalia in an effort to clasp the tip of the female's abdomen. The female did not react at first, but after about 15 s and several attempts by the male, it raised its abdomen and extended the tip backwards. The male moved to the copulatory position, and the genitalia were engaged. During copulation, the pair was motionless except for a slow pulsation in the male abdomen. The forelegs of the male rested on the sides of the thorax of the female, behind the wing bases, while the mid and hind legs grasped the abdomen (Figure 5). Copulation lasted for about 5 min, then the male genitalia were retracted, and the very long aedeagus was slowly pulled out, with repeated tractions of the whole abdomen. Still mounted on the motionless female, the male groomed its abdomen with the posterior legs, except for the genitalia,. During this time, the genitalia, with the still everted aedeagus, moved rhythmically back and forth. Slowly, over the course of about 2 min, the aedeagus was completely withdrawn to its resting position. The male groomed itself for several more minutes, still atop the female, then very suddenly the pair separated and the flies started running quickly around the vial.

A pair of *M. pellucida* was observed *in copula,* at 09:30 on 25 May 2001, perched on a *Taraxacum* flower. The male arrived suddenly and immediately grasped the female that was standing on the flower head. After a few seconds, the female raised her abdomen and extended her genitalia; at the same time, the male tilted backwards, lowering his abdomen engaging the female's genitalia; then the male resumed the horizontal position on the female's dorsum (Figure 5). The fore and mid legs were in contact with the eyes and the thorax, respectively, of the female, while the hind legs grasped the base of the female's abdomen. At intervals, the male moved his mid legs quickly and rhythmically back and forth on the female's thorax. After about 4 min, copulation was interrupted and genitalia were retracted.

Observation was terminated at this time, and the pair was collected.

As indicated above, no females of *M. picta* or *Z.* *cinereum* were observed at the site during the course of the study.

### Other observations

Observations of male activity at perch sites included the grooming behaviour of *D. aculeata.* Males (*n* = 12) were observed grooming the head, the wings, and the abdomen, genitalia included. Eyes, antennae and the extended proboscis, were repeatedly rubbed in sequence with the patch of pubescence on the inner side of both fore tibiae. As soon as the grooming was done, the tibiae were cleaned with both of the fore tarsi. The wings and abdomen were groomed by the hind tibiae, usually both wings together, then the abdomen, upper and under surfaces, and finally the everted genitalia. Also in this case, the tibiae were then cleaned by the hind tarsi. There was some degree of flexibility in the grooming movements, as a male was once observed grooming simultaneously one side of the abdomen with the respective hind leg and the opposite wing with the other leg, then alternating the sides. Grooming of the head and of wings plus abdomen, could be performed in sequence or, as in several instances, separately.

Netted *M. picta* assumed a characteristic posture (Figure 5), remaining stiff and motionless for many seconds. This posture, coupled with the disruptive patterning of the body and wings of this conopid, is very effective in concealing the fly from view. This behaviour appears to be typical of this species and has never been observed by the authors in other *Myopa.*

On Colle Vescovo, two *D. aculeata* males were found on 5 May 2003 with triungulines of a blister beetle, *Meloe* (*Eurymeloe*) sp. (M. Bologna del), attached at the base of the proboscis (see Freeman 1966).

## Discussion

Hilltopping is considered to be a strategy that facilitates mate-finding in insect species that are either rare and widely dispersed or dependent on sparsely distributed resources (Thornhill and Alcock 1983, 1987; Skevington 2008). Many species of Conopidae have very sparse populations and are very rarely seen, and more research may show that all but the most common conopids are engaged at least to some extent in hilltopping activity.

*D. aculeata* is one of the most rarely encountered conopids in Italy. In the past 14 years only five isolated specimens were collected away from hilltops in Central Italy, and, in all, only 16 records are available from Italy for the last 150 years (Mei, unpublished data). The present data indicates, however, that this species can be readily, and consistently, observed on Colle Vescovo. The two hilltopping species of *Myopa* in Italy, though being by no means “common” species, are found more often than *D. aculeata. M. pellucida,* in particular, is one of the most frequently recorded species of the genus in Italy (Mei 2000; and unpublished data) and in Europe (Stuke and Clements 2008).

Of the 19 conopid species found in the Ottawa area, only two might be considered common (Table 2). The Canadian National Collection contains 59 specimens of *P. furcillata* from over 30 sites in the region. Interestingly, only five specimens of this species have been taken on local hilltops, suggesting that it is either not a hilltopper or that it may use hilltopping only as a mating strategy in rare instances, perhaps when population densities are low. *Z. intermedium* follows a similar pattern, although another possible explanation for this species is given below. Despite being commonly encountered on hilltops, *M. clausa, P. marginata,* and *P. sagittaria* have rarely been collected away from hilltops.

The data presented above demonstrate, for the first time, the occurrence of hilltopping behaviour in 22 species of conopids. Although hilltopping in some species is represented by only a few specimens, the behaviour was pronounced and could be clearly demonstrated in *D. aculeata, M. picta, M. pellucida, M. clausa, P. marginata, P. sagittaria,* and *Z. cinereum.* The species *D. nigriceps, M. vesiculosa, Z. fulvifrons,* and *Z. intermedium* have been collected on hilltops in the past, but not exclusively so. Also, it is almost entirely males of these species that have been collected on hilltops. These specimens may represent instances of faculative hilltopping in years of low population density (Skevington 2008).

On the hilltops, males aggregated at locations that were species-specific and were occupied year after year with great consistency (e.g. Wood 1987, 1996; Alcock and Kemp 2006; Skevington 2008). On the summit of Colle Vescovo, the four species had clearly separate perch sites, i.e., the *S. junceum* and *Olea* bushes for *D. aculeata* and the relatively sheltered spot by the rock for the *Myopa* species. Species of *Myopa* on Mount Rigaud were likewise found to choose perches close to the ground. The males were almost invariably found at their specific “mating stations” (Chapman 1954; Wood 1987) during the 5-year period of the study. Similarly, the Nearctic *P. marginata* was very specific in its site choice and was rarely encountered away from a single bush. *D. nigriceps,* on the other hand, may represent an instance of aggregation location shifting due to human interference.

Males of *D. aculeata* and of *M. picta* pursued almost every flying insect of suitable size and chased conspecific males, often starting elaborate flying “contests” (e.g., *D. aculeata*). Pseudo-copulation between males was also frequently observed. These interactions could be interpreted as aggressive, territorial behaviour such as that seen in the tachinid species, *Leschenaultia adusta* (Loew) (Alcock and Kemp 2006). However, considering the whole of the available evidence, it is more convincing to exclude the occurrence of true male territoriality, according to the interpretation given by Wood (1987) for the Tachinidae. Several males (up to five in *D. aculeata*) often perched on the same bush at a very short distance from one another and did not defend their perches from conspecifics. They were never observed to actually drive away a conspecific, instead, after most of the contests, both individuals returned to their waiting stations on the same bush. Chases and pseudo-copulations may be explained as a product of heightened physical response and little discriminating ability, in the context of a strategy aiming to intercept any and all potential mates, before all other waiting males. This can be effectively done by chasing indiscriminately each and every passing insect (Thornhill and Alcock 1983; Wood 1987).

Among truly hilltopping insects, the females have been rarely encountered on hilltops, probably because they frequent the summit singly and only for the time necessary to select a mate. As a consequence, copulations are rarely observed (e.g., Chapman 1954; Dodge and Seago 1954; Alcock 1987; Wood 1987; Hansen 2003; Alcock and Kemp 2006). The present study is no exception, with females of 13 species never observed on hilltops.

The mating behaviours of *D. aculeata* and *M. pellucida* are described and illustrated here for the first time. In the latter species, the behaviour of the mating pair appeared to be almost identical to that of *Myopa buccata* (see de Meijere 1912).

Hilltopping appears to be an important mating strategy in the conopids. More research is required to understand why some species hilltop and other species do not. It may be that nonhilltoppers are more likely to attack colonial Hymenoptera. Remaining near such a colony thus is more likely to ensure mating success than hilltopping. Species of conopids that have rare or less predictably distributed hosts may be more prone to using hilltopping as a mating strategy. For example, among the European species of *Dalmannia, D. punctata* is known to mate near the colonies of their host bee (Knerer 1973) and has not been found on hilltops.

At least in some insect species, hilltopping is only one of the mating strategies available to males (Alcock 1987), and surely this is the case for the species considered here. The senior author (unpublished data) has collected *M. pellucida* pairs *in copula* on flowers in a meadow and also observed a *D. aculeata* male chasing a megachilid bee (Megachilidae) on flowers of *Silybum marianum* (Asterales: Asteraceae), in the very place and circumstances (Rome, Tenuta della Cervelletta, in the first week of May) where two females of the same species were found in preceding years. This suggests that a mating strategy alternative to hilltopping in *D. aculeata* may be used in some circumstances.

The synonymy of *Zodion notatum* with *Z.* *cinereum* has been proposed on morphological grounds (Mei and Stuke 2008). They stated that the problem of the identity of *Z. notatum* deserves a more thorough study, with a full analysis of the variability and ecology of both forms in all parts of their range. In this respect, it is interesting to note that all of the hilltopping Italian *Zodion* observed in the present study were of the *“notatum”* form, while the *“cinereum”* form was collected mainly on the slopes of Colle Vescovo and was observed on the summit only once.

*Z. fulvifrons* and *Z. intermedium* have many variant forms and ranges covering the entire southern half of the Nearctic region (Camras and Hurd 1957). The present data indicates that both species are often, but not exclusively, collected on hilltops in the Ottawa area (Table 2). These two may represent species complexes. Further study may help to unravel the taxonomy of these taxa by showing that some of these forms hilltop while others do not. Research on the systematics and ecology of conopids will benefit from future efforts that focus on hilltop collecting and observation.

Eight species of conopids were collected on Colle Vescovo (Table 1), while 13 species were collected on the summit of Mount Rigaud (Table 2). This partly emphasizes the significance of these hilltops but also suggests that many more species from other regions will be found hilltopping if the effort is made to document them. It also illustrates how important hilltops are when conducting a survey of parasitoid flies in a region. Furthermore, a loss of hilltop habitats to human development will likely endanger many rare insect species.
